# Using Protein Fingerprinting for Identifying and Discriminating Methicillin Resistant *Staphylococcus aureus* Isolates from Inpatient and Outpatient Clinics

**DOI:** 10.3390/diagnostics13172825

**Published:** 2023-08-31

**Authors:** Ayman Elbehiry, Eman Marzouk, Ihab Moussa, Sulaiman Anagreyyah, Abdulaziz AlGhamdi, Ali Alqarni, Ahmed Aljohani, Hassan A. Hemeg, Abdulaziz M. Almuzaini, Feras Alzaben, Adil Abalkhail, Roua A. Alsubki, Ali Najdi, Nawaf Algohani, Banan Abead, Bassam Gazzaz, Akram Abu-Okail

**Affiliations:** 1 Department of Public Health, College of Public Health and Health Informatics, Qassim University, Al Bukayriyah 52741, Saudi Arabia; 2 Department of Bacteriology, Mycology and Immunology, Faculty of Veterinary Medicine, University of Sadat City, Sadat City 32511, Egypt; 3 Department of Botany and Microbiology, College of Science, King Saud University, Riyadh 11451, Saudi Arabia; 4 Family Medicine Department, King Fahad Armed Forces Hospital, Jeddah 23311, Saudi Arabia; 5 Medical Director Office, North Area Armed Forces Hospital, King Khalid Military City 39747, Saudi Arabia; 6 Respiratory Therapy Department, Armed Forces Hospital Dhahran, Dhahran 34641, Saudi Arabia; 7 Patient Affairs Department, Sharourah Armed Forces Hospital, Sharourah 68372, Saudi Arabia; 8 Department of Medical Technology/Microbiology, College of Applied Medical Science, Taibah University, Madina 30001, Saudi Arabia; 9 Department of Veterinary Medicine, College of Agriculture and Veterinary Medicine, Qassim University, Buraydah 52571, Saudi Arabia; 10 Department of Food Service, King Fahad Armed Forces Hospital, Jeddah 23311, Saudi Arabia; 11 Department of Clinical Laboratory Science, College of Applied Medical Science, King Saud University, Riyadh 11433, Saudi Arabia; 12 Northern Area Armed Forces Hospital, King Khalid Military City 39748, Saudi Arabia; 13 Consultant Forensic Medicine, Forensic Medicine Center, Madina 42319, Saudi Arabia; 14 Support Service Department, King Fahad Armed Forces Hospital, Jeddah 23311, Saudi Arabia; bmriza@kfafh.med.sa; 15 Patient Affairs Department, King Fahad Armed Forces Hospital, Jeddah 23311, Saudi Arabia

**Keywords:** MALDI-TOF MS, identification, differentiation, methicillin-resistant *Staphylococcus aureus*, methicillin-sensitive *Staphylococcus aureus*, healthcare-associated infections

## Abstract

In hospitals and other clinical settings, Methicillin-resistant *Staphylococcus aureus* (MRSA) is a particularly dangerous pathogen that can cause serious or even fatal infections. Thus, the detection and differentiation of MRSA has become an urgent matter in order to provide appropriate treatment and timely intervention in infection control. To ensure this, laboratories must have access to the most up-to-date testing methods and technology available. This study was conducted to determine whether protein fingerprinting technology could be used to identify and distinguish MRSA recovered from both inpatients and outpatients. A total of 326 *S. aureus* isolates were obtained from 2800 in- and outpatient samples collected from King Faisal Specialist Hospital and Research Centre in Riyadh, Saudi Arabia, from October 2018 to March 2021. For the phenotypic identification of 326 probable *S. aureus* cultures, microscopic analysis, Gram staining, a tube coagulase test, a Staph ID 32 API system, and a Vitek 2 Compact system were used. Matrix-assisted laser desorption/ionization time-of-flight mass spectrometry (MALDI-TOF MS), referred to as protein fingerprinting, was performed on each bacterial isolate to determine its proteomic composition. As part of the analysis, Principal Component Analysis (PCA) and a single-peak analysis of MALDI-TOF MS software were also used to distinguish between Methicillin-sensitive *Staphylococcus aureus* (MSSA) and MRSA. According to the results, *S. aureus* isolates constituted 326 out of 2800 (11.64%) based on the culture technique. The Staph ID 32 API system and Vitek 2 Compact System were able to correctly identify 262 (80.7%) and 281 (86.2%) *S. aureus* strains, respectively. Based on the Oxacillin Disc Diffusion Method, 197 (62.23%) of 326 isolates of *S. aureus* exhibited a cefoxitin inhibition zone of less than 21 mm and an oxacillin inhibition zone of less than 10 mm, and were classified as MRSA under Clinical Laboratory Standards Institute guidelines. MALDI-TOF MS was able to correctly identify 100% of all *S. aureus* isolates with a score value equal to or greater than 2.00. In addition, a close relationship was found between *S. aureus* isolates and higher peak intensities in the mass ranges of 3990 Da, 4120 Da, and 5850 Da, which were found in MRSA isolates but absent in MSSA isolates. Therefore, protein fingerprinting has the potential to be used in clinical settings to rapidly detect and differentiate MRSA isolates, allowing for more targeted treatments and improved patient outcomes.

## 1. Introduction

Methicillin-resistant *Staphylococcus aureus* (MRSA) is an infection found in hospitals and other healthcare settings, where it can spread quickly among patients and cause serious, even deadly, infections [1,2]. Several types of human infections have been linked to MRSA, including minor cutaneous infections and serious systemic diseases [3,4]. Hospital-associated MRSA (HA-MRSA) outbreaks have been reported in patients who have previously been hospitalized and have suffered from clinical complications [5,6,7]. Infections caused by MRSA can be managed more effectively if clinicians detect it early and prescribe the appropriate antibiotics. Antibiotic-resistant strains, such as MRSA, have presented significant challenges to treatment [8,9]. Apart from being one of the most common causes of infectious diseases in humans, MRSA is also a pathogen that significantly impacts animal welfare due to increased drug resistance and costs associated with it [10,11]. Vancomycin-resistant *S. aureus* has only been diagnosed in a few isolated cases; however, it has not spread or established a permanent presence in healthcare facilities [12,13,14]. Without understanding the social and cultural influences on decision-making, it will be difficult to develop effective and long-lasting antimicrobial management programs. This is because social and cultural norms can shape how people perceive the use of antimicrobials and their willingness to engage in antimicrobial stewardship [15].

As a general rule, standard procedures take from 12 to 24 h for culturing the sample, followed by another couple of days for identifying the microbial species and susceptibility testing for antibiotics [16,17,18]. Researchers frequently perform Kirby Bauer tests, E-tests (Epsilometer tests), broth macros, and microdilutions to identify phenotypic characteristics [19,20,21]. Physiochemical factors such as nutrition media, pH, temperature, and solubility can influence the outcomes of these methods, though they can be time-consuming [16]. Consequently, a microbial culture assessment should be completed within 48 to 72 h of receiving the samples. Alternatively, genotypic techniques, such as polymerase chain reaction (PCR) and quantitative PCR (qPCR), reduce the duration of the incubation period. Despite the *mecA* PCR method being able to detect MRSA, the price and quality of the testing are dependent on the proficiency of the personnel [22]. Furthermore, a number of strains of methicillin-sensitive *Staphylococcus aureus* (MRSA), as well as coagulase-negative staphylococci (CNS) carrying the *mecA* gene, may also be misclassified as MRSA, since these two types of bacteria can produce a protein similar to the one produced by true MRSA [23]. Automated biochemical techniques such as BD Phoenix and ViTEK 2 Compact (bioMérieux, Craponne, France) can speed up processing times and produce more consistent results [24,25]. However, these approaches may not be feasible for small clinics with limited resources due to their cost [26].

Therefore, it is imperative to develop a reliable, quick, cheap, and affordable method of distinguishing between various types of bacteria in hospital settings. Matrix-assisted laser desorption/ionization time-of-flight mass spectrometry (MALDI-TOF MS) in medical and healthcare diagnostics is considered one of the most significant steps towards early diagnostic methods since it provides rapid and efficient discrimination of a variety of bacteria [17,27,28]. MALDI-TOF MS, a breakthrough technique for recognizing and categorizing diverse microbes, is based on protein complexes of bacterial cells [29,30]. Due to its ability to quickly complete the detection of species [31], this method has gained popularity as a detection method. Its quickness and affordability are two of its main advantages, provided there is access to a library of spectrum data covering all microorganisms [17]. Recently, applications of MALDI-TOF and machine learning approaches have been used in order to quickly distinguish MRSA from MSSA without the use of Antibiotic Susceptibility Testing (AST) approaches to diagnose antibiotic resistance.

It has been reported that Tang and his colleagues have identified nine distinct MRSA peaks in 214 *Staphylococcus aureus* (*S. aureus*) isolates [32]. Another study conducted by Liu et al. [33] showed that 452 clinical *S. aureus* isolates had 38 distinctive peaks when analyzed using a categorization algorithm. Similarly, Wang et al. used a similar approach for the analysis of 4858 mass spectra, identifying 200 peaks as features that were used for the development of a predictive model combining MALDI-TOF and machine learning techniques [34]. Elbehiry and his colleagues [28] used MALDI-TOF mass spectrometry to differentiate MRSA from MSSA by means of single peak intensities. It was observed that MRSA showed greater peak intensities in the mass ranges of 3993 Da, 4121 Da, and 5845 Da, whereas MSSA did not display those intensities.

Some researchers have used the *PSM-mec* gene, which is associated with methicillin resistance, as well as other MRSA-related unique peaks [35,36,37,38], to distinguish certain MRSA strains from others. However, despite its potential, there is a lack of data regarding multidrug-resistant bacteria in healthcare settings, and local knowledge does not support adequate research or antibiotic choices. Consequently, this study aimed to apply MALDI-TOF mass spectrometry technology for the detection and characterization of MRSA isolated from inpatient and outpatient clinics.

## 2. Materials and Methods

### 2.1. Ethical Statement

The study did not involve human participants, so ethical approval or written consent was not required. The clinical strains used in this study were obtained from routine medical testing or from strain collections, following preliminary identification by laboratory technicians at the hospital.

### 2.2. Samples and Strains

A total of 326 strains of *S. aureus* were recovered from 2800 samples collected from the King Faisal Special Hospital and Research Center in Riyadh, Saudi Arabia, between October 2018 and March 2021. These samples included 750 from upper respiratory tract infections, 800 from wound infections, 550 from lower respiratory tract infections, and 700 from skin and soft tissue infections. The isolates were transferred to the microbiology lab at Qassim University in Saudi Arabia. All isolates were sub-cultured at 37 °C for 24 h on blood agar supplemented with 5% sheep blood and MacConkey agar media.

### 2.3. Examination by Direct Microscopy

To identify isolated strains of *S. aureus* from a medium, it was necessary to examine the shape of the colonies. In the case of colonies that appeared creamy or yellow in color, this indicated success. In brief, the bacteria are first isolated on a suitable culture medium (Thermo Scientific™ Oxoid™ Baird-Parker Agar, Fisher Scientific, Göteborg, Sweden) and incubated. Then, they are prepared for viewing on a microscope slide by fixing and staining. Finally, the slide is examined under a microscope to identify the bacteria. Typically, *S. aureus* was detected as a Gram-positive organism with a blue or purple stain, presenting as small, spherical cocci or short chains, and most commonly as grape-like clusters.

### 2.4. Identification of Phenotypic Characteristics

#### 2.4.1. Staph ID 32 API System and VITEK 2 Compact System

Staph ID 32 (BioMerieux, Craponne, France) was also used according to the method described by Renneberg et al. [39] to identify the staphylococcal species found in the obtained samples. It comprised a strip containing 32 cupules, 26 of which were dehydrated biochemical media used for colorimetric testing. Based on a previously described method, all strains were also identified using the automated VITEK 2 Compact System [40].

#### 2.4.2. Coagulase Test

The tube coagulase test is an effective method of separating *S. aureus* from other Micrococcaceae [41]. This test works by causing the plasma to coagulate into a gel when coagulase is released by *S. aureus*. Briefly, the test was conducted in sterile glass tubes containing 0.5 mL of rabbit plasma (Becton, Dickinson & Company, Franklin Lakes, NJ, USA) reconstituted with a bacterial culture of three to five colonies. The coagulase plasma was gently mixed and the mixture was incubated at 37 °C for two hours. Clotting patterns were observed at 30 min intervals for the first four hours. Positive and negative control cultures were also tested to confirm the performance of the coagulase plasma technique.

### 2.5. A Standard Method for Detecting MRSA Strains

#### 2.5.1. Oxacillin and Cefoxitin Disc Diffusion Method

All strains were tested on Mueller–Hinton agar plates containing 1 mg of oxacillin and 30 mg of cefoxitin (Hi-Media, Kennett Square, PA, USA). To determine the viability of each strain, a bacterial suspension calibrated at 0.5 McFarland was used. Following an incubation period of 24 h at 35 °C, the zone of inhibition was identified. Based on clinical laboratory standards, the zone size for oxacillin is as follows: 13 mm for susceptible; 11–12 mm for intermediate; and 10 mm for resistant. For cefoxitin, the sizes were 22 mm for susceptible and 21 mm for resistant.

#### 2.5.2. CHROMagar™ MRSA

It is now possible to identify MRSA using CHROMagar™ (Hi-Media, Kennett Square, PA, USA), a new chromogenic medium. Every strain was prepared using a McFarland-adjusted suspension of bacteria at 0.5 McFarland. An earlier dipped cotton swab was used to streak the suspension onto CHROMagar™ plates. Therefore, any green colony in the presence of MRSA was considered a positive sign [42].

### 2.6. Proteomic Identification of MRSA Using MALDI-TOF MS

Bruker Daltonik, Bremen, Germany, provided our microbiology laboratory with the device for rapidly and precisely identifying all MRSA strains. The evaluation of all samples was conducted using FlexControl and Compass Flex Series Version 1.3 software. This software was used to analyze the data from each sample and compare it to a predetermined set of standards, as well as to look for trends in the data, such as changes over time. Isolates with scores greater than 2.000 should be identified at the species level, while those with scores between 1.700 and 1.999 should be identified at the genus level. The 5% Sheep Blood Agar (Sigma-Aldrich, St. Louis, MO, USA) was used to prepare the samples for MALDI-TOF MS analysis by growing them there for 18 to 24 h at 37 °C.

#### 2.6.1. Ethanol/Formic Acid Extraction Protocol

Based on the guidelines provided by Bruker Daltonik, an ethanol/formic acid extraction was performed [43]. Briefly, two to three fresh colonies were placed in a clean Eppendorf tube, and 300 µL of distilled water (DW) was mixed thoroughly with them. In the following step, 900 µL of pure ethanol was added, and the tubes were thoroughly mixed. The tubes were then inserted into a centrifuge and accelerated to 13,000 rpm for two minutes. After discarding the supernatant, the pellets were dried in air. A mixture of 50 µL of 70% formic acid and the pellets were then dissolved in 50 µL acetonitrile. One µL of the supernatant was then transferred to a stainless steel plate and allowed to dry at 25 °C after a further centrifugation at 13,500 rpm for two minutes. Each isolate was treated with one µL of a matrix solution (HCCA, α-cyano-4 hydroxycinnamic acid). The MALDI targeting plate was then attached to the Microflex LT apparatus in order to automate the run and generate the data. It was necessary to test each sample twice in order to improve the accuracy of identification. A positive control was used throughout the experiment using the Bacterial Test Standard (*Escherichia coli*).

#### 2.6.2. Discrimination, Clustering, and Data Analysis

We calculated the score value of the unidentified spectrum based on a comparison with the recognized spectrum maintained in the reference library. The validation of the strain identification was carried out by Bruker Daltonik, Bremen, Germany. MBT accurately detected both the species- and genus level, with score values ranging between 2.00 and 2.29 and 1.700 and 1.999, respectively. With a score of 0 to 1.69, the proof of identity did not meet the requirements. A wide range of spectra, from 2000 Da to 20,000 Da, were generated with the MBT Compass software, which has the capability to generate a wide range of spectra. This capability is useful for obtaining a more comprehensive understanding of the molecular structure of a sample, as different spectra will reveal different types of information about the sample. The MALDI-TOF MS was used to develop Principal Component Analysis (PCA) and single peak intensities in order to determine the difference between MRSA and MSSA isolates. The use of PCA and single peak intensities enabled researchers to look at both the overall protein composition and the specific proteins expressed by the bacterial strains. The Main Spectra Library (MSP) dataset was generated by constructing a network of pairwise distances between the subspecies. The resulting graph was then used to construct a dendrogram, which is a tree-like diagram that shows the hierarchical relationships between the subspecies.

## 3. Results

### 3.1. The Incidence of MRSA Isolation from Various Samples Obtained from Inpatient and Outpatient Clinics

**Figure 1:**
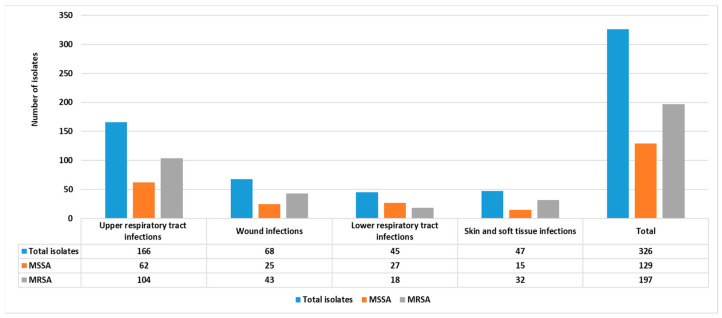
The frequency of isolation of MRSA from various samples obtained from inpatient and outpatient clinics.

According to the culture technique, 326 (11.64%) *S. aureus* isolates were identified out of 2800 samples. As shown in Figure 1, 166/326 (51%) were isolated from upper respiratory tract infections, with 62 (37.5%) MSSA and 104 (62.5%) being MRSA. Additionally, 68/326 (20.85%) were isolated from wound infections, with 25 (36.77%) being MSSA and 43 (63.23%) being MRSA. Furthermore, 45/326 (13.8%) isolates from lower respiratory tract infections were identified, with 27 (60%) being MSSA and 18 (40%) being MRSA. Finally, 47/326 (14.28%) isolates from skin and soft tissue infections were identified, with 15 (33.33%) being MSSA and 32 (68.67%) being MRSA. Based on these results, it was concluded that 197/326 (60.43%) of all samples were MRSA, while 129 (39.57%) were MSSA.

### 3.2. Identification of S. aureus Strains Based on Their Phenotypic Characteristics

For the phenotypic identification of *S. aureus*, the tube coagulase, Staph ID 32 API System, and Vitek 2 Compact System were used. Of the 326 isolates tested, 320 (98.15%) tested positive for tube coagulase and were subsequently classified as *S. aureus*. The other six strains were retested and further identified as *S. aureus*. The Staph ID 32 API system and Vitek 2 Compact System were able to correctly identify 262 (80.7%) and 281 (86.2%) *S. aureus* strains, respectively.

### 3.3. Testing for MRSA Strains Based on Standard Practices

According to the results, 197 (60.23%) out of 326 *S. aureus* isolates exhibited an inhibition zone of less than 21 mm for cefoxitin and less than 10 mm for oxacillin. According to the guidelines of the Clinical Laboratory Standards Institute (CLSI), they were classified as MRSA.

### 3.4. Protein Fingerprinting Identification of S. aureus Isolates

Analysis revealed approximately 20 prominent ion peaks in the original bands from the zone, ranging from 3000 to 15,000 Daltons (Da) in length. Among the highest peaks, the intensity was concentrated between 3785 and 6890 Da, which were synchronized with four reference isolates of MRSA stored in the Compass software library (*S. aureus* DSM 4910, *S. aureus* ATCC 33591 THL, *S. aureus* DSM 3463, and *S. aureus* DSM 20232) and five reference strains of MSSA (*S. aureus* ATCC 29213, *S. aureus* ATCC 25923, *S. aureus* DSM 20231, *S. aureus* DSM 346, and *S. aureus* DSM 799).

MSSA and MRSA isolates were correctly identified as shown in Table 1, with log scores ranging from 2.30 to 3.00 for 69 MSSA isolates and 99 MRSA isolates, respectively. Additionally, 56 MSSA isolates and 98 MRSA isolates were appropriately documented, with log values fluctuating between 2.00 and 2.29. However, the results of the analysis revealed four strains of MSSA, whose scores ranged from 1.70 to 1.99. These MSSA and MRSA strains were detected by comparing their spectra to the MBT device database, which consists of more than 300 strains of 16 genera from the ATCC and German Collection of Microorganisms and Cell Cultures GmbH (DSMZ).

**Figure 2:**
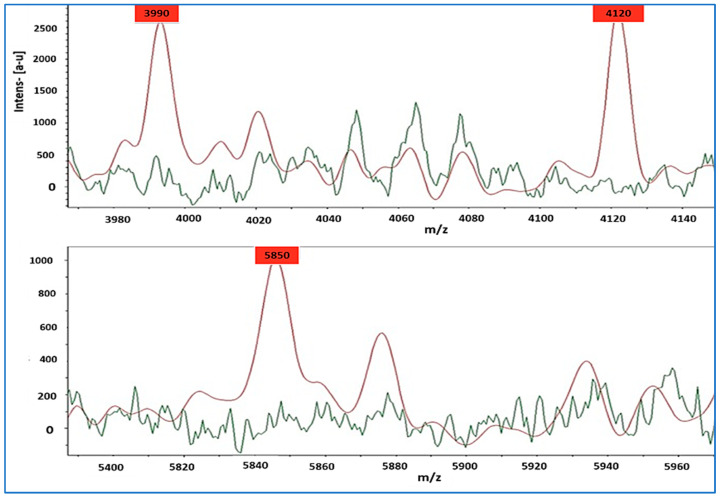
MRSA (shown in red) exhibited peaks of intensity at 3990 Da, 4120 Da, and 5850 Da, whereas MSSA (shown in green) did not display such peaks.

Based on the single-peak analysis of different mass regions, it was revealed that differences in the single-peak analyses could be used to differentiate between the strains of MSSA and MRSA. We observed numerous single peak signals in the range of 3990 to 5850 Da (Figure 2), suggesting that MSSA and MRSA strains differ greatly in their intensity levels. The MRSA (red color) patterns contained four single peak intensities located at 3990 Da, 4120 Da, and 5850 Da, whereas MSSA (green color) patterns did not contain these single peak intensities.

**Figure 3:**
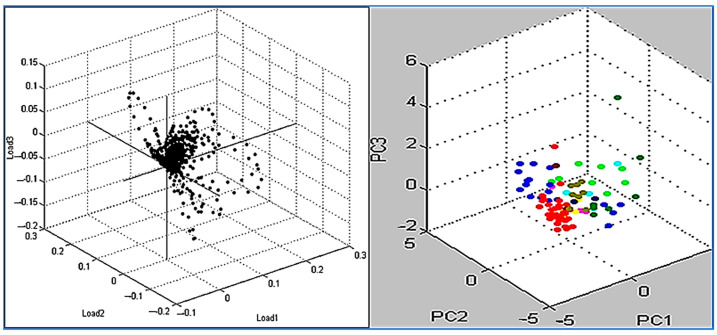
The PCA dimensional image represents several spectra for *S. aureus* isolates recovered from clinical samples. The spots represent the strength of a single signal, with the signal strength altered up to the loading value consistent with loadings 1, 2, and 3.

**Figure 4:**
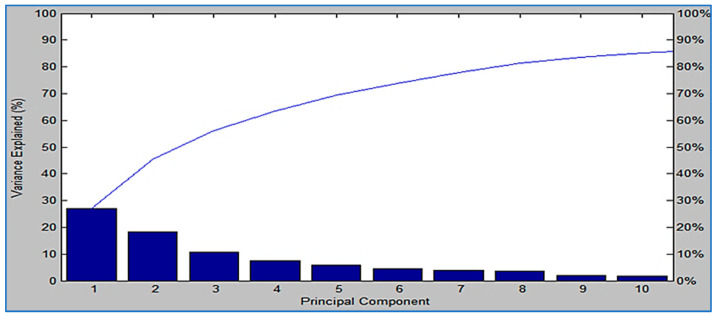
*S. aureus*’s three principal components of the PC model (PC1, PC2, and PC3) affected the proportion of explained variance by approximately 26%, 17%, and 12%, respectively.

As part of the Compass software of the MBT, the PCA was applied as a data analysis tool to show the degree of similarity and diversity in protein profile spectra. Numerous algebraic evaluations have shown that PCA also reduces the complicity of a database’s variability. Figure 3 shows that three-dimensional (3D) PCA identified a number of spectrum proteins for isolates of MSSA and MRSA. The spectrums were represented with dots, and there is a precise representation of each spectrum in the color scheme. The difference between the spectrums is shown by how each dot represents a different spectrum when viewed from the protein side. The PCA calculation set has a high probability of producing loading values generated by the PCs. There was considerable distinction between the samples from the pre-established groups due to PC1, PC2, and PC3. These principle components were also useful in displaying dimensional relationships between samples. Since PCA provided loading values, it was simple to select the contributing peaks for additional analysis. In order to calculate PCs, the variables (peaks) were loaded differently depending on how much variance they explained in the PC. Based on the calculation of PC1, PC2, and PC3, we identified each signal with loading 1, loading 2, and loading 3 values. The loading values ranged from −1 to 1, depending on their contribution to the explained variance of a PC. For *S. aureus*, it was estimated that the influences of the three principal components of the PC model (PC1, PC2, and PC3) were approximately 26%, 17%, and 12%, respectively, in the proportion of explained variance (Figure 4).

**Figure 5:**
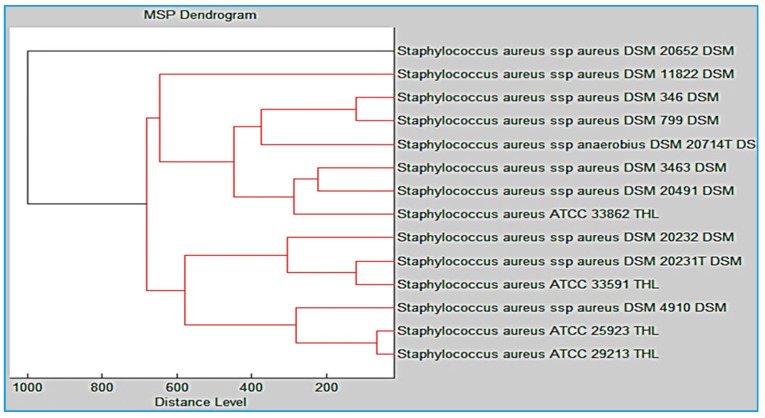
MSP dendrogram for 326 clinical isolates of *S. aureus*, matched against 14 reference strains stored in Bruker’s library.

In order to characterize the clonal lineages of *S. aureus* in MALDI-TOF MS, we analyzed 326 strains of MRSA and MSSA. We then created a cross-wise MSP dataset based on the produced spectra, as shown in Figure 5. Our findings demonstrated that the MSP dendrogram analysis revealed that the *S. aureus* strains examined were closely related to fourteen reference strains belonging to the same genus of bacteria.

## 4. Discussion

The threat posed by antibiotic-resistant bacteria has been gaining attention in recent years from several health organizations [44,45]. The spread of multidrug-resistant bacteria in hospital settings and in the general population has been the subject of numerous studies [46,47,48,49]. Among hospital and community-associated MRSA strains, which are resistant to practically all beta-lactam antibiotics and increasingly resistant to non-beta-lactam antibiotics, they have emerged as a very serious hazard [50,51,52]. The detection of MRSA in clinical samples is most commonly performed using conventional culture-biochemical and susceptibility-testing techniques. However, these methods require a considerable amount of time and effort (two to three days) [53]. As a result, nucleic acid amplification tests, such as PCR-based techniques, are sought to identify MRSA with a high degree of precision and speed [54]. Due to the requirements of specialized tools and qualified personnel [53,55], they can be difficult to use as point-of-care treatments.

In this study, 326 isolates of *S. aureus* from inpatient and outpatient clinics were identified using the Vitek^TM^ 2 Compact System. According to earlier studies, *S. aureus* strains from blood cultures could be identified with the VITEK 2 system [56,57,58]. As reported by Spanu and his colleagues, 95.6% of *S. aureus* strains recovered from blood stream infections could be properly identified using the VITEK 2 system [56]. A further study conducted by Elbehiry et al. [28] found that the Vitek^TM^ 2 compact system correctly identified 92.42% and 93.18% of MRSA and MSSA isolates, respectively. Our investigation found that the VITEK 2 System detected staphylococci with a similar degree of accuracy. With low discrimination and identification results, a very small number of strains were included in order to gain useful information to improve the VITEK 2 System; however, none of the problematic reactions were significantly more prevalent than others for the misidentified strains. This indicates that the VITEK 2 system was not properly discriminating among the different strains, and the identification results are unreliable. In some strains, slow metabolism led to ambiguous results in the reaction wells, resulting in the lack of identification of *S. aureus* [59,60].

A screen agar method based on oxacillin and cefoxitin was used for identifying all 197 MRSA in this study, showing 100% sensitivity and 100% specificity and demonstrating fantastic results. Similarly, a study by Chambers [61] reported that this method had close to 100% sensitivity for detecting MRSA. However, this method is labor-intensive and requires costly consumables such as diffusion plates and broth tubes, and takes longer to obtain results, as samples must be incubated for up to 24 h, making it less rapid than other methods.

There have been various alternatives to traditional MRSA investigation methods developed in recent years, such as chromogenic media, PCR tests and, most recently, MALDI-TOF MS [17]. The detection of MRSA is classified based on performance and effectiveness criteria, as well as convenience and efficiency criteria [62]. In comparison to qPCR approaches, which only require a few hours to complete, PCR has the advantages of high performance and efficacy [55,63]. Alternatively, MALDI-TOF MS, which is already widely used in many microbiology testing facilities, may be a useful method for detecting MRSA. The emergence of bacteria resistant to antibiotics that may be utilizing MALDI-TOF MS is attracting a growing amount of interest [62,64]. As part of the MALDI-TOF MS method for identifying pathogens, mass spectra are examined that reveal specific molecular fingerprints of germs, primarily proteins [65,66,67]. According to MSP, which is a collection of signals used to identify *S. aureus*, they account for approximately half of the total number of signals [68]. Our experiment revealed that a significant proportion of cellular proteins were highly consistent and reliable for recognizing *S. aureus*.

In less than two minutes, using the peptide mass fingerprinting method, the isolates of Staphylococcus species achieved higher scores (2.0) than conventional methods, indicating a number of advantages including a reduction in the duration of the test, making it less expensive, and an accuracy of >99% [67,68]. Other studies demonstrated the ability of MBT to successfully identify 95–100% of *S. aureus* isolates with elevated values [28,69,70]. The analysis of data obtained from the current study with MBT revealed that the majority of the spectral peaks of the tested *S. aureus* strains fell within the range of 2000 to 15,000 Da. The range of results presented in this study is consistent with those reported in previous studies involving the use of MBT for microbe identification [28,71,72]. Earlier investigations [73,74] showed that most spectral peaks fell within the range of 800 to 3500 Da, though this range was not fully reflected in the current study. This variation in mass and charge ranges of the spectral peaks may be attributed to changes in the sampling method preparation.

Previous studies have confirmed that the MBT profiles produced by MRSA and MSSA are correlated. In this study, the MALDI-TOF MS technique was successfully employed to separate MRSA from MSSA strains by identifying novel peaks that could be distinguished. Specifically, three peaks were detected at 3990 Da, 4120 Da, and 5850 Da, thus providing substantial support for the fact that MRSA and MSSA can be distinguished from one another. According to Edwards-Jones et al. [73], seven MRSA clinical isolates and seven MSSA reference strains were analyzed for *m*/*z* ranges between 500 and 10,000, and these strains showed typical spectra of 2454 Da and 3045 Da, which are common in MRSA but absent from MSSA. Moreover, Jackson et al. [75] reported that an analysis of mass spectra of MRSA showed distinct peak intensities at *m*/*z* 3048, *m*/*z* 3086, and *m*/*z* 3124. Drake et al. [76] also suggested that peaks at *m*/*z* 2302 and *m*/*z* 3871 indicate discrimination. This method yielded results in a shorter time than conventional or molecular approaches. Previous studies have proposed a methodical categorization based on these peaks in hospital studies, but this categorization is not always easily accessible. Furthermore, the reported characteristic peaks vary [17,28,77,78] or are limited to a particular subset of MRSA strains [22,37,79]. However, the discriminatory methods discussed here are highly predictable, accurate, and can be applied routinely in clinical practice.

From the previously mentioned data, MALDI-TOF MS is capable of identifying the most closely related species of organisms commonly found in clinical laboratories; however, some limitations have been observed. Possibly, MALDI-TOF’s inability to distinguish between related species may be due to the underlying similarity of the organisms themselves [67,80]. As an example, MALDI-TOF MS is not currently capable of distinguishing between *Shigella* and *Escherichia coli*; based on taxonomists’ suggestions [81], these may actually be one species, rather than two. Even so, some have suggested that MALDI-TOF MS may be able to differentiate between these microbial species [82,83]. An incomplete database of spectra may also result in identical species being mistakenly identified. This limitation presents the possibility of receiving a false species-level identification or no identification at all. In one study, it was found that similar Trichophyton species are often misidentified [84]. In addition, incorrect identification can occur if only some members of a species complex are included in the database, but not all. Body et al. observed that most isolates were correctly identified as *Mycobacterium mucogenicum*, while *Mycobacterium phocaicum*, which does not appear in the database, was frequently misidentified [85]. MALDI-TOF MS also has a limitation, currently, of being unable to fully identify polymicrobial infections directly in blood cultures [86,87]. The strong variations between different species also limit MALDI-TOF-MS’s ability to detect mixed cultures (e.g., contamination) in liquid cultured bacterial biomass. This is because MALDI-TOF-MS relies on using unique peaks to identify different species, and different species can have vastly different mass spectra [88]. Additionally, since different species can have vastly different growth rates, it can be difficult to detect contamination in mixed cultures. However, DNA microarrays allow researchers to quickly analyze a large number of genes, which can provide valuable insights into a pathogen’s identity and behavior. This helps researchers to quickly identify which pathogens are causing a particular infection and which treatments may be most effective.

## 5. Conclusions

The results of the current study indicate that patients who suffered from upper and lower respiratory tract infections, skin infections, and wound infections were more likely to be infected with MRSA. MRSA and MSSA were distinguished by MALDI-TOF MS using principle component analysis and the intensity of single peaks. MALDI-TOF MS has a high level of accuracy, sensitivity, and specificity, and is able to detect and discriminate between bacterial strains in a much shorter time than traditional methods. There are, however, limitations to the traditional MALDI TOF MS analysis. An inadequate number of spectrums in the database, combined with inherent similarities between organisms, can result in poor discrimination between species. In addition, reliable identification requires considerable biomass. While some researchers recommend that a detection limit of 6 × 10^3^ CFU/spot be used, in practice this usually results in a limit of 1 × 10^5^ CFU/spot. In the future, it will be necessary to address these limitations of the MALDI TOF MS.

## Figures and Tables

**Table 1:** Determination of score values for 129 MSSA and 197 MRSA strains using MBT.

Class	Log Score Value	No. of Identified *S. aureus*	
		MSSA	MRSA
1	2.30–3.00	69	99
2	2.00–2.29	56	98
3	1.70–1.99	4	0
4	0–1.69	0	0
Total		125 (96.9%)	197 (100%)

## Data Availability

Not applicable.

## Abbreviations

MRSA (Methicillin-resistant *Staphylococcus aureus*); MSSA (Methicillin-sensitive *Staphylococcus aureus*); *S. aureus* (*Staphylococcus aureus*); MALDI-TOF MS (Matrix-assisted laser desorption/ionization time-of-flight mass spectrometry); PCA (Principal Component Analysis); HA-MRSA (Hospital-associated Methicillin-resistant *Staphylococcus aureus*); AST (Antibiotic Susceptibility Testing); MBT (MALDI Biotyper); MSP (Main Spectra Library); ATCC (American Type Culture Collection); DSMZ (Deutsche Sammlung von Mikroorganismen und Zellkulturen); *m*/*z* (mass charge ratio).
